# Production of Sustainable Textiles Using Natural Dye and Eggshell Powder on Recycled Polyester Fabric via Waterless Supercritical CO_2_ Dyeing

**DOI:** 10.3390/polym18040431

**Published:** 2026-02-09

**Authors:** İdil Yiğit

**Affiliations:** Textile, Clothing, Footwear and Leather Department, Orhaneli Vocational School of Higher Education, Clothing Production Technology, Bursa Uludağ University, Bursa 16980, Türkiye; idilyigit@uludag.edu.tr

**Keywords:** sustainability, natural dye, eggshell, recycled, waterless dyeing, supercritical carbon dioxide

## Abstract

The growing environmental impact of conventional textile dyeing processes, particularly their high water consumption, chemical usage, and wastewater generation, has intensified the need for alternatives. For this reason, the textile industry faces increasing pressure to adopt sustainable production routes that minimize environmental loads. The utilization of recycled polyester fabrics, natural dyes, and waste-derived bio-resources within waterless dyeing systems represents a holistic approach toward environmentally responsible textile manufacturing. This study focuses on the production of sustainable textiles by dyeing recycled polyester fabrics with natural madder dye and eggshell powder in a waterless supercritical CO_2_ medium. The samples were characterized via SEM, TGA, wash fastness tests, and tensile strength measurements. SEM images clearly revealed the presence of eggshell powder (ESP) on the fabric surfaces. After UV aging, the samples containing 20% ESP exhibited higher tensile strength and more pronounced color stability compared to the control sample. The CaCO_3_ component of the ESP contributed to UV resistance, while the TGA results showed higher residual mass for ESP-treated samples, indicating improved thermal stability. Moreover, the persistence of ESP on the fabric surface after repeated washing and the satisfactory wash fastness results confirmed the durability of the treatment. Overall, the results demonstrate that the combination of natural dye, recycled polyester, and eggshell-derived bio-additives in a waterless scCO_2_ dyeing system offers a promising and environmentally benign strategy for producing sustainable and functional textile materials.

## 1. Introduction

Recently, sustainability and environmentally friendly production have become important topics of discussion around the world, largely due to global warming and the rapid depletion of natural resources. As in every field, many studies related to sustainability are being conducted in the textile industry [1,2], since the textile sector is one of the main contributors to environmental pollution [3,4,5]. Achieving sustainability requires a holistic approach encompassing all production processes, including post-use recycling. To this end, numerous studies have explored the use of natural fibers and natural dyes in waterless environments [6].

Sustainability efforts are part of an ongoing process that seeks to create a cohesive whole. For example, many synthetic fiber manufacturers contribute by producing recycled polyester from waste PET and textile waste, thereby reducing production costs and carbon emissions [7,8]. Dye manufacturers also support sustainable and environmentally friendly policies by producing natural-based dyes derived from plant extracts, minerals, or animal sources, which are biodegradable, renewable, and environmentally friendly, or through new-generation eco-friendly synthetic dyes and auxiliaries [9,10,11].

Another sustainability initiative in textiles involves minimizing or eliminating water consumption during production. Waterless dyeing using supercritical carbon dioxide (scCO_2_) is an important method for reducing textile wastewater. ScCO_2_ is inexpensive, non-flammable, eco-friendly, and chemically inert under various conditions. Additionally, it can be easily removed from the system by depressurization. This technique has been applied both in laboratory-scale studies and commercially, particularly in the dyeing of virgin and recycled polyester fabrics [12,13,14].

The roots of *Rubia tinctorum* L., the Latin name of madder, serve as a natural source of dye, typically containing various anthraquinone-based compounds, including *alizarin, purpurin, munjistin, pseudopurpurin, and nordamncanthal* [15]. Previous studies have reported the use of madder root for dyeing synthetic fibers such as polyester in both aqueous (wet) and waterless dyeing media [16,17]. Although alizarin, a compound derived from *Rubia tinctorum* L., has been applied successfully to dye PET and recycled PET fabrics in supercritical CO_2_ systems, the present study differs by investigating the direct use of madder root in combination with eggshell powder (ESP) for polyester dyeing in scCO_2_, representing a novel material combination that has not yet been reported.

The primary objective is to support environmentally friendly production by establishing production stages that are as close as possible to sustainability [13,14,18,19]. Eren et al. [15] and Haji et al. [16] investigated the dyeability of polyester fabrics with natural dyes in a scCO_2_ environment and stated that this approach offers a promising alternative to conventional high-temperature, high-chemical polyester dyeing methods [15,16].

Another important aspect of sustainable production is waste management. Waste can be converted into valuable and useful resources, improving both sustainable development and adequate waste management strategies. Eggshells are a typical example of product-specific waste in the food processing industry, with utilizable parts still present in waste [20,21]. Global egg production is expected to increase by 50% by 2035. This means that the waste generated after egg consumption will increase by the same percentage. The Environmental Protection Agency states that egg waste ranks 15th in the waste hierarchy of the food industry [22]. Waste eggshells are generated daily from the consumption of eggs in households and industrial concerns. With time, the increasing volume of waste eggshell generated and discarded indiscriminately has attracted the attention of environmentalists and researchers to investigate how the waste can be converted into useful products. The eggshell consists mainly of calcium carbonate (~94%), magnesium carbonate, calcium phosphate, and organic matter, and accounts for 10% of the total egg weight. Traditionally, without any form of modification, eggshells have been used in many different industries such as: agriculture and animal husbandry, food and household applications, and construction and building materials [23]. Beyond the aforementioned application areas, the utilization of unmodified eggshells has also been explored within the context of textile-related applications. To date, there have been limited reports on the direct application of eggshell powder in textile dyeing processes. Most existing studies focus on the use of eggshell powder as an adsorbent for dye removal in wastewater treatment [24,25,26], rather than as an active component in dyeing baths. As such, no comprehensive studies have been published that apply eggshell powder directly in the dyeing of textile substrates. In contrast, the present study investigates the combination of madder and eggshell powder for polyester dyeing, representing a novel approach not reported in the previous literature.

Previous studies involving eggshell powder in textile-related contexts have predominantly focused on environmental applications rather than direct textile dyeing processes. For instance, Ngadi et al. [24] investigated eggshell powder as a low-cost adsorbent for the removal of methylene blue dye from aqueous solutions, demonstrating effective dye adsorption governed by contact time, adsorbent dosage, and initial dye concentration. Similarly, Azhar and Rashid [25] reported the use of eggshell waste for the adsorption of both heavy metals and dyes from textile wastewater, achieving high removal efficiencies and highlighting its suitability for wastewater treatment applications. In addition, Ghaneian et al. [26] evaluated eggshell as a natural sorbent for the removal of Reactive Red 123 dye from synthetic textile wastewater, showing adsorption efficiencies of up to approximately 80% under optimized conditions. Notably, all these studies address dye removal from aqueous media for environmental remediation purposes, rather than the direct use of eggshell powder within textile dyeing baths or during the dyeing of textile substrates.

At the same time, eggshells have been reported to enhance materials’ mechanical, thermal, and UV (ultraviolet) protective properties [21,27,28,29]. In particular, Fecheyr-Lippens et al. [27] investigated the use of unprocessed chicken eggshells as UV-protective additives for synthetic polymers (polystyrene and nylon), demonstrating that eggshells can provide durable and effective protection against ultraviolet radiation, highlighting their potential for UV-protective applications in material systems.

Tseghai et al. [28] applied a flame-retardant treatment to cotton fabrics using calcium carbonate and components with flame-retardant properties, such as phosphorus, nitrogen, potassium, and zinc, contained in eggshell powder (ESP). The results revealed that fabrics treated with ESP exhibited lower flammability compared to untreated samples.

In Megna’s [30] study, the feasibility of using waste eggshell powder as an eco-friendly and sustainable filler/carrier material in textile printing pastes was investigated. The study examined the role of ESP on the rheological properties, print quality, and surface effects of the printing paste. The results showed that ESP could serve as an alternative to traditional inorganic fillers while maintaining the clarity of the print pattern, offering a sustainable approach to textile printing.

The main motivation of this study is to dye recycled polyester fabrics using the natural dye madder in combination with ESP at different ratios, without the use of any additional chemicals, in a scCO_2_ environment. Eggshells, which are an abundant bio-waste rich in calcium carbonate (CaCO_3_), are incorporated into the dyeing system due to their potential to act as a natural, inorganic functional additive. In this context, ESP is expected to contribute to dye fixation and color stability through its mineral structure, while simultaneously enhancing performance properties such as color fastness, UV protection, and thermal stability. By integrating recycled polyester, a natural dye, and a waste-derived bio-resource into a single, solvent-free dyeing process, this approach aims to valorize eggshell waste, reduce chemical consumption, and develop an innovative and sustainable dyeing strategy.

In the present work, we explore the use of eggshell powder in combination with madder dye for the dyeing of polyester fabric, which, to the best of our knowledge, represents a novel application in this field. This approach differs from previous literature by assessing the functional role of eggshell powder in enhancing dye uptake and color performance on a synthetic fiber system, rather than in environmental remediation contexts.

## 2. Materials and Methods

### 2.1. Materials

In this study, a 100% recycled polyester plain fabric (100 g/m^2^) was used. The madder root (*Rubia tinctorum* L.) dye was sourced as a finely ground powder from a local natural dye store located in Ardakan, Iran. White chicken eggshells used as a support material were supplied by the Veterinary Department of Bursa Uludağ University, Bursa, Türkiye. For the waterless dyeing process’s scCO_2_ media, we employed the Rapid Xiamen ModelH-12 oil bath dyeing machine in conjunction with specialized vessels from DyeCoo (Weesp, The Netherlands), which is situated at Bursa Uludağ University’s Textile Engineering Laboratory.

### 2.2. Methods

#### 2.2.1. Processing of Chicken Eggshells

**Figure 1:**
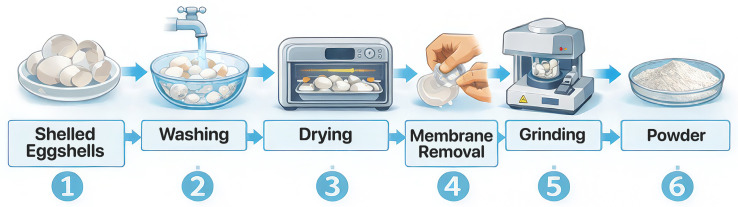
Eggshell powder production steps. 1. Shelled eggshells, 2. Washing, 3. Drying, 4. Membrane removal, 6. Grinding, 7. Powder.

The collected chicken eggshells were thoroughly washed individually under running water. Eggshells from raw eggs were used for ease of removing the membranes. The eggshells were soaked overnight for ease of removing both the inner and outer membranes. The eggshells were then dried for 5 days at room temperature (20 ± 5 °C). The flow chart of the process is given in Figure 1.

The cleaned and dried eggshells were mechanically ground using a high-speed rotary grinder (in-house) to obtain a fine powder. After grinding, the eggshell powder was sieved using 325 mesh. The size of the ESP particles was in the low micrometer range (~1–3.0 µm).

#### 2.2.2. Application of Natural Dye and ESP to Fabric

**Figure 2:**
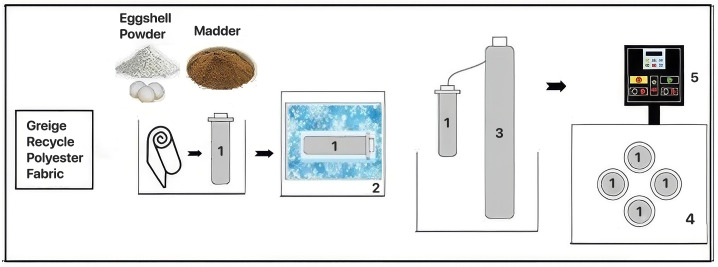
ESP and natural dye madder application process steps in scCO_2_. 1. Dyeing vessel, 2. Freezer, 3. CO_2_ tank, 4. Oil (Polyethylene glycol) bath, 5. Control panel.

The dyeing procedures were conducted within the waterless scCO_2_ dyeing machine. supercritical CO_2_ (scCO_2_) exhibits combined gas-like diffusivity and liquid-like density when operated above its critical temperature and pressure. ScCO_2_ acts both as a solvent for the dye and as a diffusion-enhancing medium. CO_2_ molecules penetrate the amorphous regions of polymeric fibers, inducing temporary swelling and increasing free volume within the structure. This plasticization effect facilitates dye transport and diffusion into the fiber matrix while reducing processing time. After treatment, CO_2_ is completely depressurized and removed from the system, eliminating the need for solvent recovery or drying steps [6]. For a visual representation of the scCO_2_ dyeing machine, please refer to the schematic image provided by Yiğit et al. 2021 [12]. Each recycled polyester fabric sample weighed 5 g, while the most available dyestuff amounted to 20% owf (on weight of fabric) [15]. The recycled polyester fabric samples were placed in the vessel. Finely powdered madder was used as the sole dye source for the control sample, while ESP was incorporated at 5, 10, and 20% owf in the experimental samples. The vessel was filled with CO_2_. The amount of CO_2_ needed for each sample was calculated according to the *NIST Chemistry Webbook* based on the specified pressure and temperature. For easy filling of CO_2_, the vessels were cooled in a separate freezer for 15 min and mounted on the pattern rotating inside the dyeing oil bath. Since the main objective of this study was to investigate the properties of eggshell powder after its use in the dyeing process under supercritical carbon dioxide conditions, no further optimization of dyeing parameters was required. The dyeing parameters were selected based on the conditions reported by Eren et al. (2024), in which the best results were obtained through controlled experiments (118 °C, 20 MPa, and 73 min) [15]. The produced samples were rinsed with warm water after the process. The application is shown in Figure 2.

#### 2.2.3. Scanning Electron Microscope (SEM) Analysis

A high-resolution Carl Zeiss AG-EVO^®^ 40 Series SEM (Jena, Germany) device was used under low vacuum to observe the presence of ESP on the surfaces of the samples and for the EDS analyses of the ESP. To ensure optimal conductivity and minimize charging effects during imaging, all samples were coated with gold and palladium, using a Leica EM ACE200 (Vienna, Austria) device. Images were taken at different magnification levels to clearly show the processes applied to the samples.

#### 2.2.4. Thermogravimetric Analysis (TGA)

Thermogravimetric analysis (TGA) was performed on a Shimadzu DTG-60H (Shimadzu DTG-60H (Kyoto, Japan) by heating from room temperature to 600 °C at a rate of 10 °C min^−1^.

#### 2.2.5. UV Aging Procedure

UV treatments were carried out in a specially designed UV cabinet equipped with UV lamps positioned on both sides of the chamber. The UV cabinet was operated under standard laboratory atmospheric conditions (20 ± 2 °C temperature and 65 ± 4% relative humidity). The system contained 18 UV-C lamps (254 nm, Philips, Hamburg, Germany) with a total installed power of 470 W. During the experiments, only the side lamps were operated, providing a total UV power of 254 W (127 W per side).

The fabric sample was suspended at the center of the cabinet, resulting in a fixed distance of 21 cm between the UV lamps and the sample surface. Samples were exposed to continuous UV irradiation for 168 h under controlled conditions, following an accelerated aging approach reported in the literature [31].

This in-house UV aging protocol was designed based on the general principles of ISO 105-B02 [32], ISO 4892 [33], and ASTM G154 [34] standards.

* ASTM G154 Standard Practice for Operating Fluorescent Light Apparatus for Exposure of Nonmetallic Materials, American Society for Testing and Materials, West Conshohocken, Pennsylvania, USA. to simulate accelerated photo-oxidative aging. The temperature inside the UV cabinet was monitored using a thermometer placed within the chamber throughout the exposure period. No active temperature control was applied, and the cabinet temperature remained close to ambient conditions, with any temperature increase resulting solely from UV lamp operation.

After UV exposure, tensile strength and color change were evaluated to assess the effects of UV-induced degradation.

#### 2.2.6. Tensile Strength Test 

Tensile testing was performed according to ISO 13934-1 [35] on a Shimadzu Model AG-X-Plus Tensile Tester (Kyoto, Japan) under standard laboratory conditions.

#### 2.2.7. Color Differences Test 

Color difference (ΔE) is a metric that represents the color difference between two samples. The ΔL, Δa, and Δb values of the samples were measured (representing the differences in lightness (ΔL), red–green axis (Δa), and yellow–blue axis (Δb) between two colors, respectively) via a Konica Minolta CM3600D (Tokyo, Japan) spectrophotometer under illuminant D65 using a 10° standard observer. ΔE is a metric that represents the color difference between two samples. The equation for the ΔE value is shown below (Equation (1)).

ΔE*ab = √((ΔL*)^2^ + (Δa*)^2^ + (Δb*)^2^) (1)

#### 2.2.8. Washing Fastness Test

The washing fastness tests of dyed samples were performed via a Test Laboratory Instrument (model 412 NB HT, Turkiye) according to the ISO 105-C06-A1S [36]. The staining on the multifiber test fabric was evaluated objectively via a Konica Minolta CM3600D spectrophotometer.

In order to observe the particles of ESP, the process was repeated 5 times. ESP particles were observed using SEM.

## 3. Results and Discussion

### 3.1. Characterization of Samples

**Figure 3:**
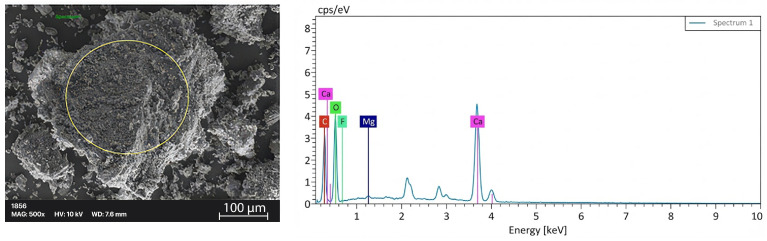
EDS spectrum of ESP (yellow-circled area).

The elemental composition of the ESP samples was analyzed by EDS (energy-dispersive spectroscopy) to evaluate the effect of increasing additive content on surface chemistry. EDS analysis revealed that the ESP mainly consisted of calcium and oxygen, confirming the calcium carbonate (CaCO_3_) structure of the material. The relatively low carbon content can be attributed to the inherent limitations of EDS in detecting light elements. The EDS spectrum of ESP is given in Figure 3, and elemental analysis of ESP is shown Table 1. 

With increasing eggshell content, a gradual increase in calcium (Ca) signal was observed, indicating successful deposition of the additive on the surface. The Ca atomic percentage increased proportionally with the additive ratio, confirming controlled and reproducible incorporation.

The madder root dye which contains *munjistin*, *alizarin*, *purpurin*, *xanthopurpurin*, and *rubiadin* [15], was used in this study. Among these compounds, *alizarin* was the major coloring compound that brought the red hue to the samples dyed with madder.

### 3.2. SEM Images

#### 3.2.1. SEM Images of Various ESP Amounts

**Figure 4:**
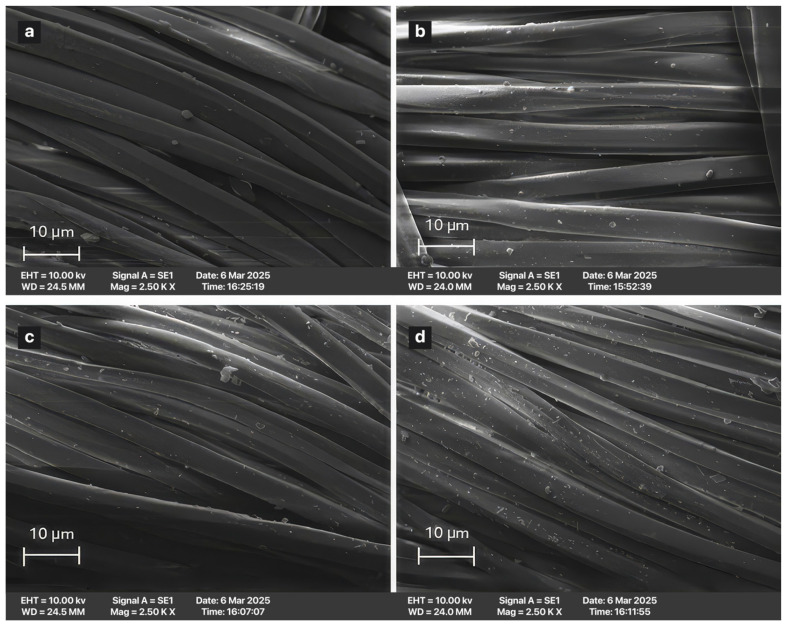
SEM images of dyed samples treated with different percentages of ESP (each image is enlarged ×2500 magnification). (**a**) Control (dyed/0%ESP). (**b**) Dyed/5% ESP. (**c**) Dyed/10% ESP. (**d**) Dyed/20% ESP.

SEM images of ESP applied at different percentages are shown in Figure 4.

ScCO_2_ acts as a plasticizer/swelling agent for polyester [37]. During the dyeing process, the distance between the polymeric chains increases, the amorphous regions expand, and the dye diffuses into the fiber [38,39]. However, in this study, the ESP particles used alongside the dye do not penetrate into the fiber structure. One of the main reasons for this behavior is particle size (~1–3.0 μm). While natural dye molecules are on the nanometer scale [40], ESP particles are in the micrometer (µm) range, making their physical penetration into the fiber structure impossible. Diffusion occurs for the dye due to its molecular-scale dimensions, whereas no diffusion is observed for the CaCO_3_ particles constituting the ESP. Consequently, although the supercritical CO_2_ environment effectively transports molecular species, it does not facilitate the diffusion of solid particulate matter. Another reason is that there are no chemical bonds in the medium. CaCO_3_ does not chemically react with ester groups of polyester; scCO_2_ acts as a non-reactive, environmentally benign solvent and transport medium. It does not dissolve ionic species such as Ca^2+^. Therefore, integration into the fiber is not possible.

Most natural dyes exhibit moderate or limited light stability compared to synthetic dyes; therefore, auxiliary agents such as mordants are commonly required to improve dye fixation and light fastness [41]. Previous studies have demonstrated that eggshell powder (ESP) can provide durable and effective UV protection owing to its mineral composition [27,42].

Under high pressure, scCO_2_ temporarily swells the polyester fiber structure, increasing free volume and surface roughness. The literature reports that during this swelling process, micro-voids may form within the fiber matrix and surface irregularities may develop. Although ESP particles are too large to diffuse into the polyester structure, these conditions allow micron-sized particles to mechanically adhere to the softened fiber surface or to remain immobilized at the fiber/scCO_2_ interface. Such stable surface adhesion of inorganic particles is rarely observed in conventional aqueous dyeing systems, as water does not facilitate the retention of particles of this size on the fiber surface [43,44,45].

Accordingly, in the present study, ESP is proposed to act not as a diffusing species, but as a surface-associated functional additive, contributing to improved UV protection and potential dye stabilization at the fiber surface rather than through molecular-level penetration.

In a scCO_2_ environment, the fiber surface softens (due to the swelling effect) and micro-roughness increases. Exposure of polyester to supercritical carbon dioxide lowers the glass transition temperature and increases the free volume by swelling the polymer’s amorphous regions, facilitating dye diffusion into the fiber [46]. In related surface-modification studies, scCO_2_ processing has been associated with alterations in fiber surface morphology, where treated fibers exhibit changes in topography compared to untreated ones, consistent with increased molecular mobility and surface restructuring during treatment [47].

#### 3.2.2. SEM Images of Repeated Washing

**Figure 5:**
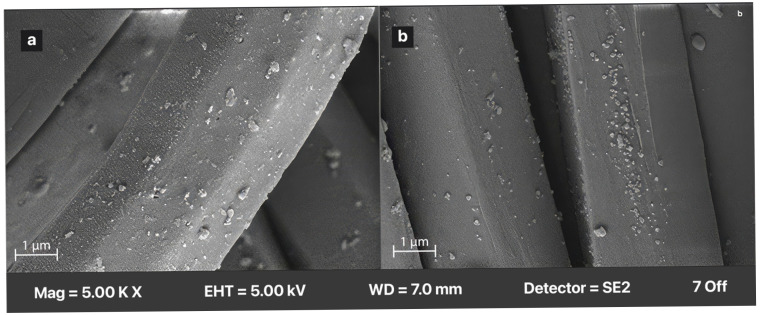
SEM images of samples treated with 20% ESP after 5 repeated washes. (**a**) Before repeated washing. (**b**) After repeated washing (×5000 magnification).

Figure 5 shows the ESP particles on the samples after five repeated washing cycles performed, washing procedures under controlled laboratory conditions were applied according to the ISO 105-C06-A1S using a standard detergent solution at 40 °C for 30 min. After washing, the samples were rinsed with distilled water and dried under ambient conditions. To observe the particles of ESP, the washing process was repeated five times. The ESP particles were observed using SEM. The ESP particles remained on the surface, but their density decreased and they aggregated compared to the pre-wash condition. The removal of particles from the surface in certain areas due to the mechanical washing effect is an expected outcome. As noted in the analysis of the SEM images, the ESP particles are located on the surface and have not penetrated the fiber structure. Therefore, a reduction of the surface or accumulation in certain areas after prolonged mechanical effects is an expected result. However, their continued presence on the surface is an important and developable feature for the sustainability of the process.

### 3.3. Thermogravimetric (TGA) Behavior of the Samples

**Figure 6:**
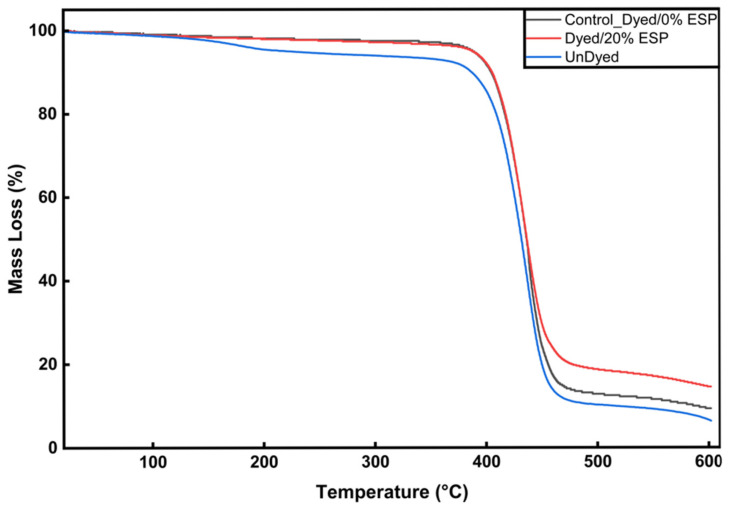
Comparative TGA graph of control dyed, dyed with 20% ESP and undyed samples.

The TGA results of the control and ESP-additive samples are given in Figure 6.

The TGA curve for the control dyed 0% ESP recycled polyester sample (black line) shows a gradual decrease in mass in the 0–350 °C temperature range, with the main decomposition (onset) occurring between approximately 390 and 440 °C. In contrast, the sample containing 20% ESP (red line) maintains a mass that is almost constant in the same temperature range, i.e., it exhibits more stable behavior at low temperatures. This indicates that the inorganic components (especially mineral/calcium compounds) and protein structure contained in the ESP limit the movement of volatile components in the polyester matrix, reducing mass loss at early temperatures. For comparison, the undyed sample (blue line) exhibits a thermal degradation profile very similar to that of the control dyed 0% ESP sample, with only minor differences observed in the initial mass loss region. This indicates that the dyeing process itself has a limited influence on the overall thermal stability of the polyester fibers, and that the changes observed in the TGA curves are predominantly associated with the presence and content of the ESP rather than dye-related components.

At 600 °C, the residual mass of the undyed sample treated with ESP is approximately 6–8%, whereas the control dyed sample without ESP exhibits a residue of about 10%. With the incorporation of 20% ESP, the residual mass increases significantly to approximately 18–20%, indicating enhanced char formation and improved thermal stability.

This increase is important for inorganic mineral content and carbon char layer formation. ESP contains high levels of mineral components (e.g., calcium salts), which are more resistant to combustion and remain in the solid phase after decomposition at high temperatures. The organic portion of ESP can contribute to the formation of a carbon-rich char layer during thermal decomposition, which partially blocks heat and mass transfer through the flame, thereby protecting the underlying polymer phase. Thus, the material exhibits higher residue retention and potentially improved flame resistance compared to raw polyester.

Due to their micron-scale particle size and inorganic crystalline nature, eggshell-derived CaCO_3_ particles cannot diffuse into the polyester fiber matrix. SEM observations indicate that the particles are mainly located on or partially embedded into the softened fiber surface. For the sample produced with 20% ESP loading, the surface coverage of ESP particles was quantified to be approximately 20%, as determined by image analysis using the ImageJ software (https://imagej.net/ij/download.html, accessed on 22 January 2026). The changes observed in the TGA results are therefore attributed to the presence of thermally stable inorganic particles and their barrier effect rather than true bulk incorporation into the fiber.

In addition to the contribution of ESP, the possible effect of dye components on the thermal behavior was also considered. To evaluate the potential influence of the dye, TGA curves of the undyed and dyed samples without ESP were also included for comparison [19,22,23].

### 3.4. Tensile Strength After UV Aging

Various thermal effects and photo-oxidative processes can cause chain decomposition reactions and the formation of new chemical bonds in the molecular structure of PET. Within the scope of these processes, chain scission due to thermal degradation between vinyl and carboxyl end groups and photodegradation of methylene groups, which causes irreversible effects, occur; these structural changes lead to a significant decrease in the mechanical strength of PET [48].

**Figure 7:**
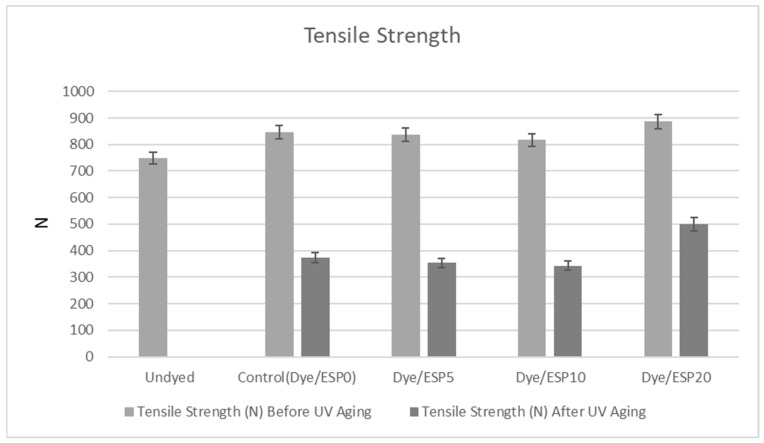
Tensile strength values of dyed samples with different percentages of added ESP before and after UV aging.

Before UV aging, the tensile strengths of undyed, dyed, and dyed with added ESP samples were comparatively evaluated, as shown in Figure 7. Compared to the undyed samples, both dyed and dyed with added ESP containing samples exhibit a moderate increase in tensile strength. As discussed in Section 3.2.1, the addition of ESP does not alter the chemical structure of the polyester fibers. The eggshell-derived CaCO_3_ particles are mechanically fixed within surface microcracks formed during fiber swelling and remain primarily on the fiber surface. Therefore, despite the increase observed after dyeing, no statistically significant difference in tensile strength is observed between dyed samples with and without ESP prior to UV aging.

After UV aging, however, a clear differentiation is observed depending on the ESP content. The control sample without ESP (ESP0) exhibits the highest UV sensitivity, showing a tensile strength loss of approximately 56%. Samples containing 5% and 10% ESP show behavior similar to the control sample after UV aging, indicating that low ESP loadings are insufficient to effectively limit UV-induced chain scission and photo-oxidative degradation.

In contrast, the sample containing 20% ESP showed the highest tensile strength after UV aging (≈499 N), with the strength loss limited to approximately 44%, corresponding to a 12% reduction in tensile strength compared to the control sample. These results clearly indicate that ESP does not enhance the tensile strength prior to UV exposure, but provides a protective effect during UV aging at sufficiently high loading levels.

At a 20% ESP ratio, CaCO_3_ particles act as a physical barrier by scattering UV radiation. In addition, CaCO_3_ particles that are mechanically attached to and partially embedded in the fiber surface under scCO_2_ conditions restrict UV penetration into the fiber bulk, thereby reducing chain scission and photo-oxidative degradation. Consequently, eggshell-derived CaCO_3_ does not function as a reinforcing filler but rather as a UV-protective inorganic surface layer, preserving the mechanical integrity of polyester fibers after aging.

### 3.5. Color Differences After UV Aging

**Figure 8:**
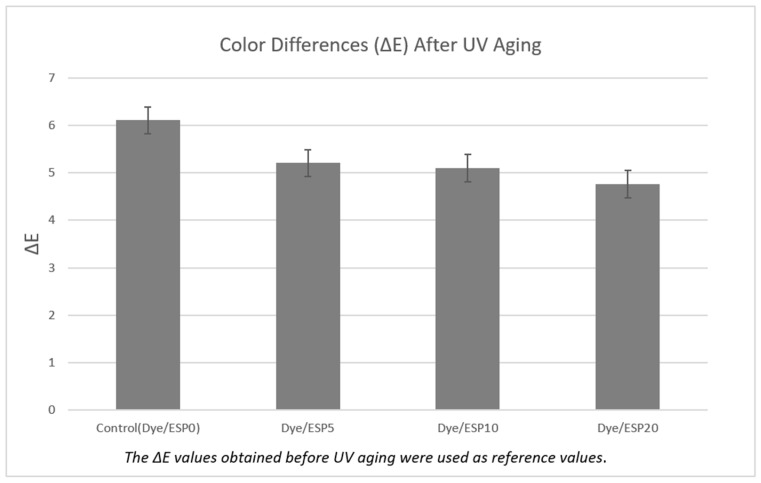
Color difference results after UV Aging.

**Figure 9:**
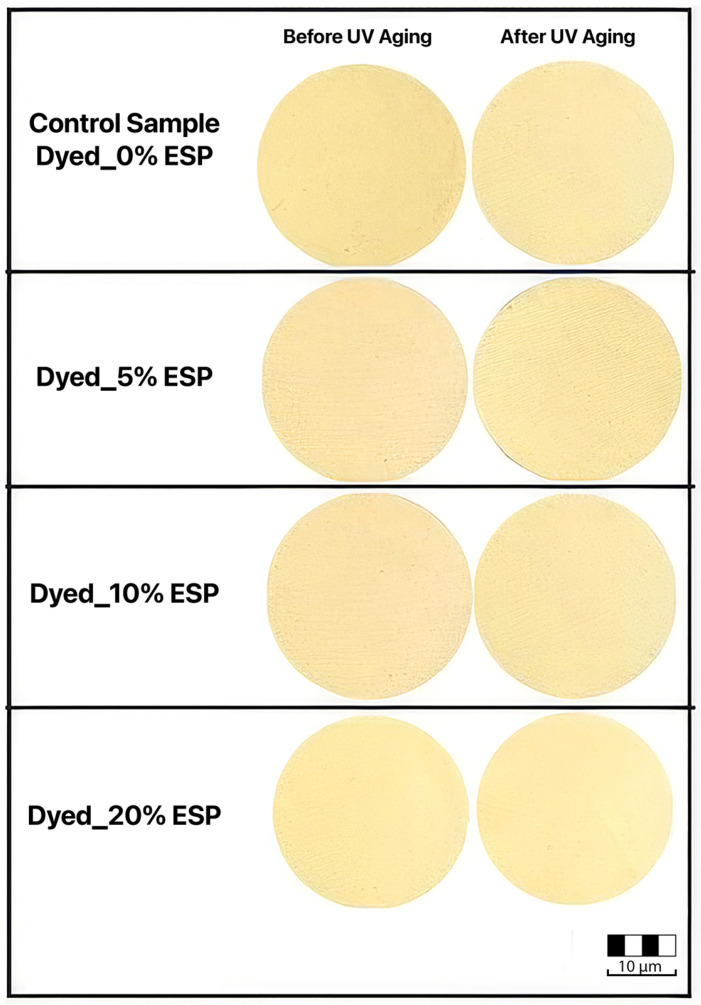
The images * of samples before and after UV aging. * The images were taken using a standard camera in a light cabinet under D65 at a fixed distance (20 cm).

The color difference data are given in Figure 8 and images are given in Figure 9. When Figure 8 is examined, it is clearly seen that the color difference (ΔE) values after UV aging show a decreasing trend depending on the ESP content. The control sample (ESP0) has the highest ΔE value (~6.1); this indicates that the natural dye degrades more under UV radiation. Natural dyes are generally known to exhibit lower lightfastness compared to many synthetic dyes, leading to faster color fading or shade changes upon prolonged light exposure. This behavior is mainly attributed to the molecular structure of natural dyes, which are often more susceptible to photodegradation and form weaker, less permanent interactions with textile fibers than engineered synthetic dyes. As a result, the color durability and long-term stability of naturally dyed textiles are inherently limited under UV exposure [27,42].

When the results in Figure 8 are examined, the control sample without ESP exhibits the highest color difference after UV aging, indicating pronounced color change compared to the original sample. The samples containing 5% and 10% ESP show lower ΔE values after UV exposure, suggesting a partial reduction in color change; however, the difference between these two concentrations is not significant. The sample containing 20% ESP exhibits the lowest ΔE value (~4.8), demonstrating the most effective reduction in color change after UV aging. These results indicate that the incorporation of ESP contributes to improved color stability by reducing UV-induced color change. The enhanced performance at higher ESP content is attributed to the UV scattering effect of CaCO_3_ particles, which limits the UV energy reaching the dye molecules and thereby reduces photodegradation.

### 3.6. Washing Fastness

Table 2 lists the washing fastness properties of the samples dyed under optimal conditions. It can be observed that all the samples exhibited excellent washing fastness (grade 4–5 to 5) on multifiber fabrics. The high fastness degree of washing of the dyed samples indicates good penetration of the dye molecules with ESP into the fibers in the presence of scCO_2_. The values obtained in the dyeing process, which is carried out using only natural dye and ESP without any auxiliary chemicals, mordants, or surface modification, are significant.

## 4. Conclusions

This study demonstrated the feasibility of producing sustainable and functional textiles by dyeing recycled polyester fabrics with natural madder dye and ESP in a waterless-scCO_2_ medium without the use of additional chemicals. The integration of ESP, a waste-derived bio-resource primarily composed of CaCO_3_, provided not only an environmentally benign route for waste valorization but also contributed to the enhancement of textile performance properties.

SEM analysis confirmed the stable deposition and retention of ESP on the fabric surface even after repeated washing, indicating good durability of the treatment. The samples containing 20% ESP exhibited lower color difference (ΔE) values after UV aging compared to the control sample, indicating reduced color change. This result demonstrates that the incorporation of ESP provides enhanced resistance against UV aging by minimizing UV-induced color degradation. In addition, the 20% ESP-containing samples showed higher tensile strength retention after UV exposure compared to the control samples, further confirming the protective effect of ESP against UV-induced degradation. The sample containing 20% ESP showed the highest tensile strength (≈499 N) after UV aging, with strength loss limited to 44%. These results reveal that high CaCO_3_ content plays a protective role in preserving the fiber structure during UV aging.

Furthermore, the TGA results revealed increased residual mass for ESP-treated samples, demonstrating enhanced thermal stability. The UV-shielding effect associated with the CaCO_3_ content of ESP effectively reduced color degradation under accelerated aging conditions.

Overall, the combination of natural dyeing, a recycled polyester substrate, waste-derived functional additives, and waterless scCO_2_ technology offers a novel and sustainable dyeing strategy aligned with circular economy principles. This approach has significant potential for reducing water and chemical consumption in textile processing while simultaneously improving functional performance, paving the way for future developments in eco-friendly and high-performance textile materials.

## Figures and Tables

**Table 1:** Elemental analysis of ESP.

Element	Mass Norm (%)
Carbon	8.37
Oxygen	38.39
Fluorine	0
Magnesium	0
Calcium	53.24

**Table 2:** Staining of multifiber after washing.

Sample	Wool	Acrylic	Polyester	Polyamide	Cotton	Acetate
Control (Dye/ESP0)	5	5	5	5	5	4–5
Dye/ESP5	5	5	5	5	5	4–5
Dye/ESP10	5	5	5	5	5	5
Dye/ESP20	5	5	5	5	5	5

Multifiber is a specialized test fabric composed of stripes of different fiber types (wool, acrylic, polyester, polyamide, cotton, acetate) used to assess dye staining after washing.

## Data Availability

The original contributions presented in this study are included in the article. Further inquiries can be directed to the corresponding author.
